# Limited Capability for Testing *Mycobacterium tuberculosis* for Susceptibility to New Drugs

**DOI:** 10.3201/eid2703.204418

**Published:** 2021-03

**Authors:** Hamzah Z. Farooq, Daniela M. Cirillo, Doris Hillemann, David Wyllie, Marieke J. van der Werf, Csaba Ködmön, Vlad Nikolayevskyy

**Affiliations:** Public Health England, London, UK (H.Z. Farooq, D. Wyllie, V. Nikolayevskyy);; San Raffaele Scientific Institute, Milan, Italy (D.M. Cirillo);; Research Centre for Mycobacteria, Borstel, Germany (D. Hillemann);; European Centre for Disease Prevention and Control, Stockholm, Sweden (M.J. van der Werf, C. Ködmön)

**Keywords:** Mycobacterium tuberculosis, bacteria, tuberculosis and other mycobacteria, tuberculosis, antimicrobial resistance, drug susceptibility, drugs, testing, public health, respiratory infections, Europe

## Abstract

We surveyed availability of phenotypic drug susceptibility testing for drug-resistant *Mycobacterium tuberculosis* in Europe. Of 27 laboratories, 17 tested for linezolid, 11 for clofazimine, 9 for bedaquiline, and 6 for delamanid during 2019. Our findings indicate that testing capacity for newer and repurposed tuberculosis drugs exists, but its availability is limited.

*Mycobacterium tuberculosis* is a major cause of death globally, and increasing predicted deaths from tuberculosis (TB) are caused by delays in diagnosis and treatment of new cases associated with coronavirus disease containment measures (*1*). Drug-resistant, multidrug-resistant (MDR), and extensively drug-resistant (XDR) TB remain major public health issues (*1*).

In the World Health Organization European Region, the proportion of rifampin-resistant and MDR TB is greater than the global average. New drug regimens incorporating bedaquiline, clofazimine, linezolid, and delamanid to treat MDR and XDR TB have been recommended by the World Health Organization and are being implemented globally (*2*). For newer and repurposed drugs (NRDs), phenotypic drug susceptibility testing (pDST) is not yet fully standardized because of a lack of data for epidemiologic cutoff values. In addition, genomic DST (gDST) lacks sensitivity, and genetic mechanisms of drug resistance have yet to be fully established for NRDs (*3*).

There have been issues with procuring pure substances for testing and availability of resistant isolates (non-XDR strains) for validation of assays. The widely used BACTEC mycobacteria growth indicator tube (MGIT) technology (Becton Dickinson, https://www.bd.com) has not been calibrated against a reference standard protocol and is not fully validated for second-line drugs, highlighting the need for sustainable external quality assessment (EQA) schemes. For well-tolerated compounds, (i.e., moxifloxacin), phenotypic and genotypic resistance prediction using current interpretive guidance might be discordant, leading to uncertainty about clinical efficacy.

Availability of pDST for bedaquiline, clofazimine, linezolid, and delamanid in Europe is unknown, which is of concern in areas that have higher incidences of drug resistance, such as eastern Europe. Within a framework of EQA schemes implemented by the European TB Reference Laboratory Network and coordinated by the European Centre for Disease Prevention and Control, we performed a survey on the availability and performance of pDST for NRDs in European Union/European Economic Area laboratories during 2018–2019.

EQA is one of the key components of the European TB Reference Laboratory Network, which uses an interlaboratory comparison to enable objectivity in auditing the performance of a laboratory (*4*). For the EQA panels, 5 well-characterized *M. tuberculosis* isolates that have varying drug susceptibility profiles, including those resistant to >1 NRDs (n = 3), were sent to participating laboratories. These laboratories were requested to test samples for phenotypic susceptibility to drugs routinely tested in their laboratory. Reports received were analyzed against reference results by using established protocols (*4*).

A total of 28 laboratories participated and reported results within the deadline of the EQA (2018–2019). In this EQA panel, results were reported for 15 drugs, including NRDs. Of 28 laboratories, 15 tested for linezolid, 6 for clofazimine, 6 for bedaquiline, and 4 for delamanid during 2018. This increased to 17 tested for linezolid, 11 for clofazimine, 9 for bedaquiline, and 6 for delamanid during 2019 (Figure). All but 1 laboratory used MGIT methods for NRDs (*5*).

**Figure:**
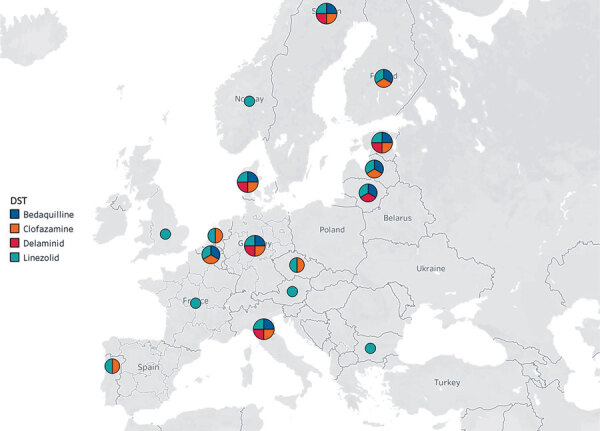
European Union laboratories performing phenotypic DST of new tuberculosis drugs, 2019. Map courtesy of Mapbox OpenStreet Map (https://www.mapbox.com). DST, drug susceptibility testing.

During 2019, a total of 17 laboratories reported 100% correct results and 9 laboratories reported 96%–99% correct results. One laboratory scored 85%, and 1 laboratory scored only 69% (i.e., below the threshold for certification). There were 3 very major errors (false-susceptible results) for clofazimine (n = 2) and bedaquiline (n = 1), and 1 major error (false-resistant result) for linezolid. Other errors included very major errors for protionamide (n = 4), isoniazid (n = 3), moxifloxacin (n = 1), and streptomycin (n = 1) and major errors for moxifloxacin (n = 3), rifampin (n = 2), isoniazid (n = 1), and protionamide (n = 1).

Our findings show that availability of pDST is increasing in Europe but remains limited. With the increasing availability of the NRDs for TB, standardized and validated pDST of *M. tuberculosis* in culture isolates to NRDs is crucial for appropriate use of new drugs in treatment regimens.

Ongoing global efforts to define a set of quality control strains and standardize MGIT methods against a reference to overcome variability (*6*) are being complemented by ensuring clear rules on when pDST is required and to ensure that gDST is fully used. Appropriate use of pDST and gDST is essential for clinical management of MDR and XDR TB to limit transmission and prevent development of further resistance (*7*). Use of bedaquiline and delamanid requires special approval in certain countries because of substantial cost of this drug (*8*). Thus, performing pDST for NRDs is essential to ensure that isolates are susceptible to the newer agents.

Validated and quality-controlled pDST, including automated liquid culture systems and microtiter plate-based assays for NRDs, might be used to improve accuracy of prediction of resistance and susceptibility to NRDs by using whole-genome sequencing (*9*). Although pDST is considered the standard for susceptibility testing for NRDs, using whole-genome sequencing will help to detect and characterize new mutations and insertions/deletions associated with drug resistance and also analyze strain relatedness rapidly, resulting in prompt public health actions, and thus will be highly useful. Use of data from multicenter EQA sites (*10*) can help develop standardized guidelines for pDSTs for global use. In addition, pDST data for isolates can be used for additional studies to validate predicting resistance to gDSTs for NRDs.
